# A multiplex real-time PCR for the detection and differentiation of *Campylobacter* phages

**DOI:** 10.1371/journal.pone.0190240

**Published:** 2017-12-22

**Authors:** Claudia Jäckel, Jens A. Hammerl, Jörg Rau, Stefan Hertwig

**Affiliations:** 1 Department of Biological Safety, German Federal Institute for Risk Assessment (BfR), Berlin, Germany; 2 Chemical and Veterinary Investigatory Office (CVUA) Stuttgart, Fellbach, Germany; University of Helsinki, FINLAND

## Abstract

*Campylobacter jejuni* and *C*. *coli* are important food-borne pathogens that are widespread in animal husbandry. To combat *Campylobacter* along the food chain, the application of lytic phages has been shown to be a promising tool. *Campylobacter* phages are currently classified into three groups, of which group II and group III phages are the most common. Members of each group are closely related, whereas the two groups share only little DNA similarity. Moreover, while group III phages are specific for *C*. *jejuni*, group II phages additionally infect *C*. *coli*. Phage cocktails intended to be used for applications should be composed of various phages that differ in their host range and growth kinetics. The isolation of phages is generally performed by plaque assays. This approach has the limitation that phages are merely identified by their lytic activity on certain indicator strains and that relatively high numbers of phages must be present in a tested sample. Therefore, a more sensitive molecular detection system would be beneficial, which allows a pre-screening of samples and the quick detection and discrimination of group II and group III phages. New phages can then be isolated by use of indicator strains that may be different from those typically applied. On the basis of available *Campylobacter* phage genome sequences, we developed a multiplex PCR system for group II and group III phages selecting the tail tube gene and the gene for the base plate wedge, respectively, as target. Phages of both groups could be identified with primers deduced from the putative tail fiber gene. Efficient release of phage DNA from capsids was achieved by an extended heat treatment or digestion of phage particles with proteinase K/SDS yielding a detection limit of 1 pfu/ml. Individual detection of group II phages, group III phages and of both groups was studied with artificially contaminated chicken skin. To recover phages that had strongly adhered to the skin, stomaching was the most efficient technique. The developed PCR protocol was employed to detect *Campylobacter* phages in food and environmental samples. In 50 out of 110 samples group II and/or group III phages were identified.

## Introduction

Campylobacteriosis is a worldwide zoonosis and the most frequent bacterial enteritis in the European Union (EU) [1]. Typical symptoms are diarrhea, cramping, abdominal pain, and fever. The disease is mainly caused by the thermophilic species *Campylobacter jejuni* (*C*. *jejuni*) and its close relative *C*. *coli*. Human infections generally occur by the consumption of undercooked meats, especially poultry [2]. *Campylobacter* is a common commensal of the gastrointestinal tract of various mammals and birds [3] and frequently found in chicken farms where the bacteria may spread rapidly [4]. To reduce the number of this pathogen in chicken and on chicken products, phages that have also been used for typing of *Campylobacter* strains [5–7] might be an appropriate means. Indeed phage administration in the laboratory reduced *C*. *jejuni* colonization of the broiler gut and the contamination on chicken skin by several orders of magnitude [1, 3, 4, 8–19]. In addition, a field trial with *Campylobacter* phages has been successfully carried out in commercial broiler flocks [20, 21].

On the basis of their genome size and morphology, the currently known lytic *Campylobacter* phages are divided into three groups [22]. While group I phages (~320 kb) have been rarely isolated and have not been used for applications yet, members of group II (~175 to 183 kb) and group III (~131 to 135 kb) are very common [22]. Group II phages have generally a broader host range than phages of group III since they frequently infect both *C*. *jejuni* and *C*. *coli* strains. Though, the successive application of a group III and a group II phage reduced the numbers of *C*. *jejuni* in chickens most efficiently [15]. What is common to group II and group III phages are a similar myoviridal morphology, a low burst size, a very low GC content of 26 to 27% and a resistance against cleavage by many restriction endonucleases, probably caused by modification of the bases cytosine or guanine [7, 23, 24]. These properties hampered the quick identification of group II and group III phages and the molecular characterization of these phages for many years. Up to now three group II (CP21, CP220 and CPt10) and eight group III (CP81, CP30A, NCTC12673, PC14, PC5, vB_CjeM_Los1, CPX and CP8) phages have been completely sequenced. The genomic sequence of group II phage vB_CcoM-IBB_35 (IBB_35) has been deposited in the form of five contigs [25]. Phages within each group revealed strong DNA homologies whereas only weak similarities exist between group II and group III phages for which two new genera, the "Cp220likevirus" and "Cp8unalikevirus", respectively, have been proposed [22, 26]. Both groups are distantly related to T4-like phages [22, 23, 27, 28]. They possess a core genome comprising genes for virion assembly and for proteins involved in replication and DNA packaging. A striking feature of group II phages is that their genomes are composed of large modules separated by long DNA repeat regions, which could lead to rearrangements [26–29]. Thus far two subgroups of group II phages exhibiting a different modular genome organization and host range have been identified [27]. *Campylobacter* phages are generally isolated by plaque assays using indicator strains (e.g. *C*. *jejuni* NCTC12662), which are susceptible to a broad range of phages [8, 9, 30, 31]. However, the choice of indicator strains may influence the finding of new phages possibly suitable for applications [32]. Although phages within each group are genetically very similar, they may differ in terms of their host range and lytic activity. Thus, a molecular approach could help to detect even low numbers of group II and group III phages quickly, which can then be isolated by use of common or uncommon indicator strains.

In this study, a multiplex real-time PCR (qPCR) system has been developed for the sensitive detection of group II- and group III-related phages. To achieve this the genomes of the hitherto described *Campylobacter* phages were searched for the presence of possible target sequences. After examination of all targets by conventional PCR, the best suited sites were selected to design primers and probes for qPCR. Individual detection of group II phages, group III phages and of both groups together was studied with spiked chicken meat. Following this, an optimized protocol was established and used to analyse food and environmental samples for the presence of *Campylobacter* phages.

## Material and methods

### Bacterial strains and phages used in this study

All strains were provided by the National Reference Laboratory for *Campylobacter* of the German Federal Institute for Risk Assessment. Cultivation of *Campylobacter* spp. was performed on blood agar Base II (Oxoid, Wesel, Germany) supplemented with 5% calf blood [15]. Phages used to design and verify the PCR are listed in Tables 1 and 2.

**Table 1:** Phage genomes analysed for the presence of possible target sequences.

*Campylobacter* phage	Accession number	Description (genome size, origin)	Group	Reference
**CP8unalikevirus**				
CP81	FR823450.1	132,454 bp, Germany	III	[23]
CP30A	JX569801.1	133,572 bp, UK	III	[33]
NCTC12673	GU296433.1	135,041 bp, UK	III	[34]
PC14	KX236333.1	134,927 bp, Slovenia	III	[35]
PC5	KX229736.1	131,095 bp, Slovenia	III	[35]
vB_CjeM_Los1	KX879627.1	134,073 bp, Ireland	III	[36]
CPX	NC_016562.1	132,662 bp, UK	III	Unpublished
CP8	KF148616.1	132,667 bp, UK	III	[33]
**CP220likevirus**				
CP21	HE815464.1	182,833 bp, Germany	II	[27, 29]
CP220	FN667788.1	177,534 bp, UK	II	[28]
CPt10	FN667789.1	175,720 bp, UK	II	[28]
vB_CcoM-IBB_35^1^	HM246720.1 to HM246724.1^1^	172,065 bp^1^, Portugal	II	[25]

^1^ The genome of vB_CcoM-IBB_35 (IBB_35) is available in five sequence contigs.

**Table 2:** Phages used to verify the developed PCR.

*Campylobacter* phage	Accession number	Description	Group	Reference
**CP8unalikevirus**				
CP81	FR823450.1	132,454 bp, Germany	III	[23]
CP1	n.a.	~135 kb*, Germany	III	[15]
CP14	n.a.	~135 kb*, Germany	III	[15]
F14	n.a.	~135 kb*, Denmark	III	[15, 30]
CP32	n.a.	~135 kb*, UK	III	[15]
**CP220likevirus**				
CP21	HE815464.1	182,833 bp, Germany	II	[27, 29]
CP75	n.a.	~185 kb*, UK	II	[27]
CP7	n.a.	~185 kb*, Germany	II	[27]
CP68	n.a.	~185 kb*, Germany	II	[27]
CP84	n.a.	~185 kb*, UK	II	[27]
CP83	n.a.	~185 kb*, UK	II	[27]

n.a.: not available;

*: genome sizes were estimated by PFGE analysis of phage DNA.

### Isolation, propagation and purification of *Campylobacter* phages

To detect phage-induced lysis, plaque and spot tests were conducted using the softagar double-layer technique [37]. For the preparation of overlay agar plates, 500 μl of bacterial cultures (OD_588 nm_ 0.9 to 1.4) were added to NZCYM soft agar (Sigma Aldrich, Taufkirchen, Germany) [27]. For phage propagation and activity tests, the following indicator strains were used: *C*. *coli* NCTC12668 (CP21, CP68, CP83, CP84), *C*. *jejuni* NCTC12662 (CP7, CP75, IBB_35, F325), *C*. *jejuni* NCTC11168 (CP1, CP14, CP32, CP81, F14) and *C*. *jejuni* RM1221 (F376-F389).

High-titre lysates of phages were obtained as previously described [27] Bacterial DNA and RNA was removed by three consecutive DNaseI (20 μg/ml) and RNaseA (20 μg/ml) treatment steps for at least 4 h at 37°C. The lysates were concentrated by ultracentrifugation and purified by CsCl step gradients [27].

### Preparation and quantification of phage DNA for PCR analyses

Phage DNA was prepared as previously described [27]. Determination of the concentration and purity of all phage DNAs was carried out by Qubit analysis according to the manufacturer´s recommendations. Results obtained with the Qubit 2.0 spectrophotometer (Life Technologies, Darmstadt, Germany) were compared with band intensities of phage DNA analysed in ethidium bromide stained 0.8% agarose gels.

### Examination of possible target sequences

Conventional PCR analyses were performed in an Eppendorf Mastercycler ep Gradient (Eppendorf, Hamburg, Germany). PCR reactions were prepared in a final volume of 25 μl using the DreamTaq DNA polymerase amplification components (Fisher Scientific, Schwerte, Germany). The mastermix of each reaction comprised 13.35 μl of RNase-free water, 2.5 μl of 10 x DreamTaq Buffer, 2.5 μl of dNTP solution (2 mM), 0.65 μl of DreamTaq Enzyme, 2.5 μl of each primer and 1.0 μl of template DNA (10–20 ng/μl). The following protocol was used: initial PCR activation and template denaturation at 94°C for 120 s followed by 35 cycles comprising a denaturation step at 94°C for 15 s, annealing at 47°C for 15 s and elongation for 60 s at 72°C. In addition, the protocol contained a final elongation step at 72°C for 1 min before the PCR reactions were stored at 4°C until further processing. If necessary, PCR products were purified using the MSB Spin PCRapace kit (Stratec, Birkenfeld, Germany). Commercial Sanger sequencing was conducted by Eurofins Genomics (Ebersberg, Germany).

### Real-time PCR detection assay

In general, real-time PCR (qPCR) runs were performed in duplicate using an ABI 7500 Fast real-time PCR System (Applied Biosystems, Darmstadt, Germany). qPCRs were performed using the Qiagen Pathogen Detection Kit including a synthetic internal amplification control (Qiagen, Hilden; Germany). Validation of the qPCR assay was performed by use of both the ABI 7500 Fast real-time PCR System and the CTX96 cycler (Bio-Rad Laboratories GmbH, Munich, Germany). Each individual reaction had a final volume of 20 μl containing 0.2 mM of forward and reverse primers, 0.3 nM of the probe (Table 3), 10 μl of Pathogen Detection Master Mix (Qiagen, Hilden, Germany) and 1 μl of purified phage DNA. The amplification protocol included an initial activation step at 95°C for 20 s followed by 40 cycles of denaturation at 95°C for 15 s and annealing at 60°C for 30 s. Threshold values were automatically generated by the 7500 Fast real-time PCR software and the Bio-Rad-CFX-Manager (v 3.1, Bio-Rad Laboratories Inc.). In each experiment, a non-template control (NTC), negative control (NC) and DNA of group II (CP21) and group III (CP81) *Campylobacter* phages (positive controls) were included. To determine the sensitivity of the detection assay, a standard curve of CP81 and CP21 phage DNA was created in triplicate for different DNA concentrations. Standard curves were generated by amplifying 1:10 DNA dilution series of 5 x 10^8^ phages. The specificity of the detection assay was determined by examination of all available *Campylobacter* phages and phages of other host bacteria (*E*. *coli*, *Vibrio*, *Klebsiella*, *Salmonella*, *Yersinia*, MRSA, etc.). For further analyses, phage lysates were used as template for real-time PCR.

**Table 3:** Primers and probes for the qPCR detection *of Campylobacter* phages.

**Oligonucleotide**	**Sequence (5’-3’)**	**Specificity (target)**
CPGII-F	TATTTTTGTCACGCTACAAGTTTT	Group II phages (CP21 ORF186)
CPGII-R	ACATTTGTTGGAAATACATTCATC	
CPGII-probe	FAM-CCGGGATTGACTGTAGAAACA-BHQ-1	
CPGIII-F	TTCAGGGATAAATGAAAACCAAA	Group III phages, (CP81 ORF008)
CPGIII-R	AGTTGGCACTGATGAAGAAACC	
CPGIII-probe	Cy5-TGTAACTGCCCTGTTTGCTG-BBQ-650	
CPGII/GIII-F^1^	TGTAGATCTTTCTAGTGGDAGTAAYG	Group II & III phages (CP21 ORF096 and CP81 ORF002)
CPGII/GIII-R^1^	ACTATTATTTYCAGAGCTKCCTTTA	
CPGII/GIII-probe	YY-TTTGGAACTAGTGCTACAAATCC-BHQ-1	

Abbreviations: F: forward primer; R: reverse primer; probe: fluorescently labeled primer; FAM: 6-Carboxyfluorescein, BHQ-1: Black Hole Quencher Dye 1, Cy5: Cyanine Dye 5, YY: Yakima Yellow, BBQ-650: BlackBerry Quencher 650, Y: C or T, K: G or T, D: G, A or T.

### Testing of the developed qPCR detection system with spiked meat samples

To evaluate the suitability of the detection assay for routine testing, natural contamination of food samples was simulated by spiking of chicken meat purchased at local supermarkets (Berlin, Germany) in January 2015. Before spiking the samples were investigated by qPCR and plaque assays for the presence of *Campylobacter* phages. Spiking was performed by adding SM-buffer containing the *Campylobacter* phages CP81 and CP21 (10^3^ to 10^9^ pfu) to the surface of the meat samples (25 cm^2^). To allow adherence of the phages to the surface of the meat, the samples were incubated for 1 h at 4°C. Meat samples without phages were used as negative controls (NCs). To ascertain by which technique the highest yield of phages added to chicken skin can be recovered, three different sample preparations were compared, (A) swab sampling, (B) homogenization of the skin and (C) flushing of chicken skin pieces (25 cm^2^). To open the pores of the chicken skin, meat samples were incubated for 30 min at 42°C after adding phage. For swab sampling, swabs were moisturized with 2 ml buffered peptone water. Three consecutive sampling steps were conducted. After each step the swabs were resuspended in 1 ml buffered peptone water. Homogenization was carried out by diluting (1:10) meat samples in buffered peptone water in sterile stomacher bags (BagPage 400 ml; Interscience, St. Nom la Bretèche, France). Stomaching was performed at the highest intensity in a BagMixer 400SW (Interscience) for 5 min. Flushing was conducted by three steps using 10 ml buffered peptone water. Aliquots of suspensions from each procedure were tested in triplicate by qPCR and plaque testing.

### Bioinformatic analyses

Sequence alignments and primer design were carried out using the Accelrys DS Gene software package (Accelrys Inc., USA) and primer 3web (v 4.0.0) [38]. Similarity and identity values were calculated using different BLAST algorithms (http://www.ncbi.nlm.nih.gov/BLAST/) at the NCBI homepage. In addition, primers already described in another study [28] have been tested.

## Results and discussion

### Identification of suitable PCR target sequences on the phage genomes

**Fig 1:**
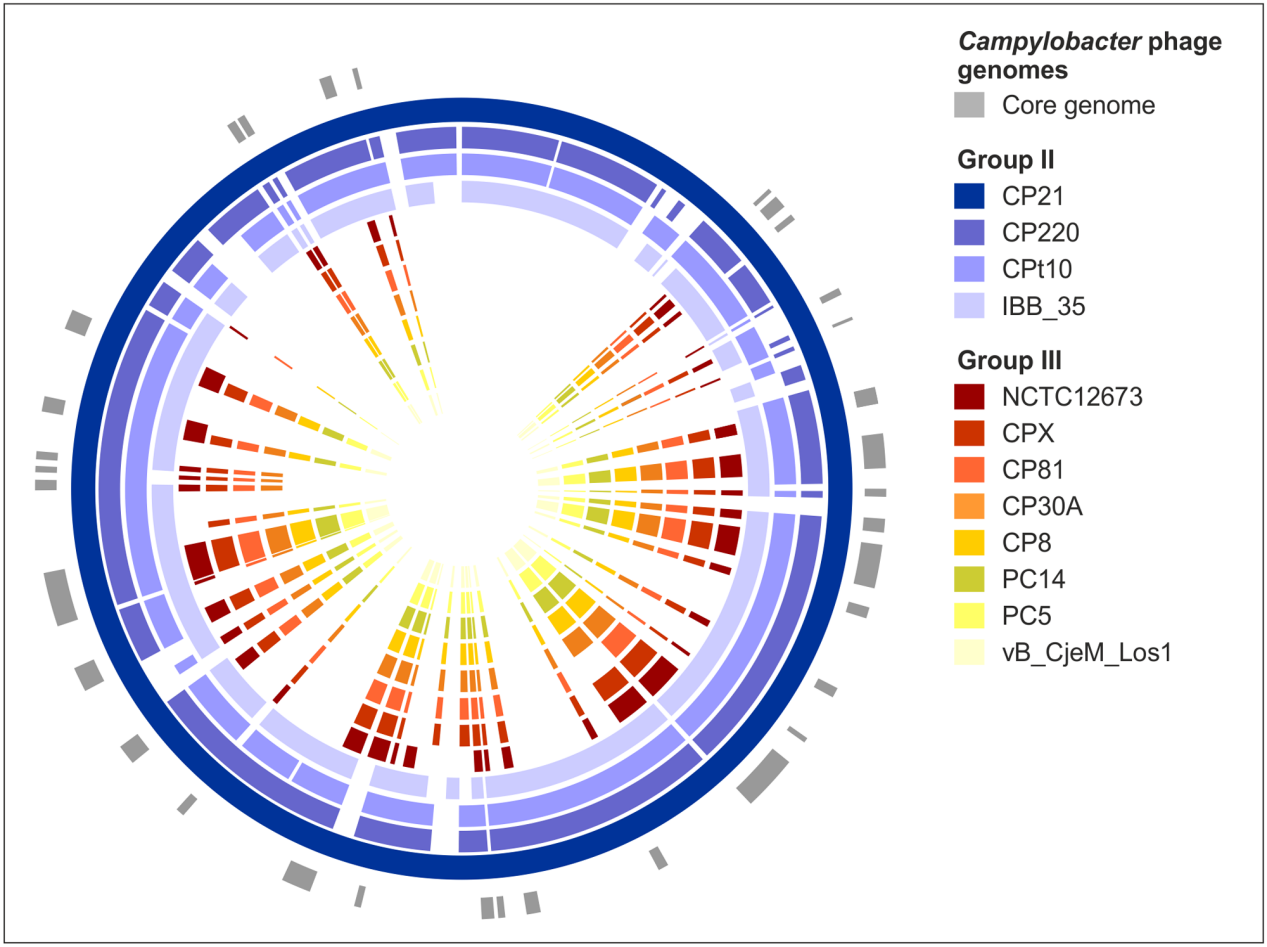
Synteny plot based on the amino acid similarity of *Campylobacter* group II and group III phages.

**Fig 2:**
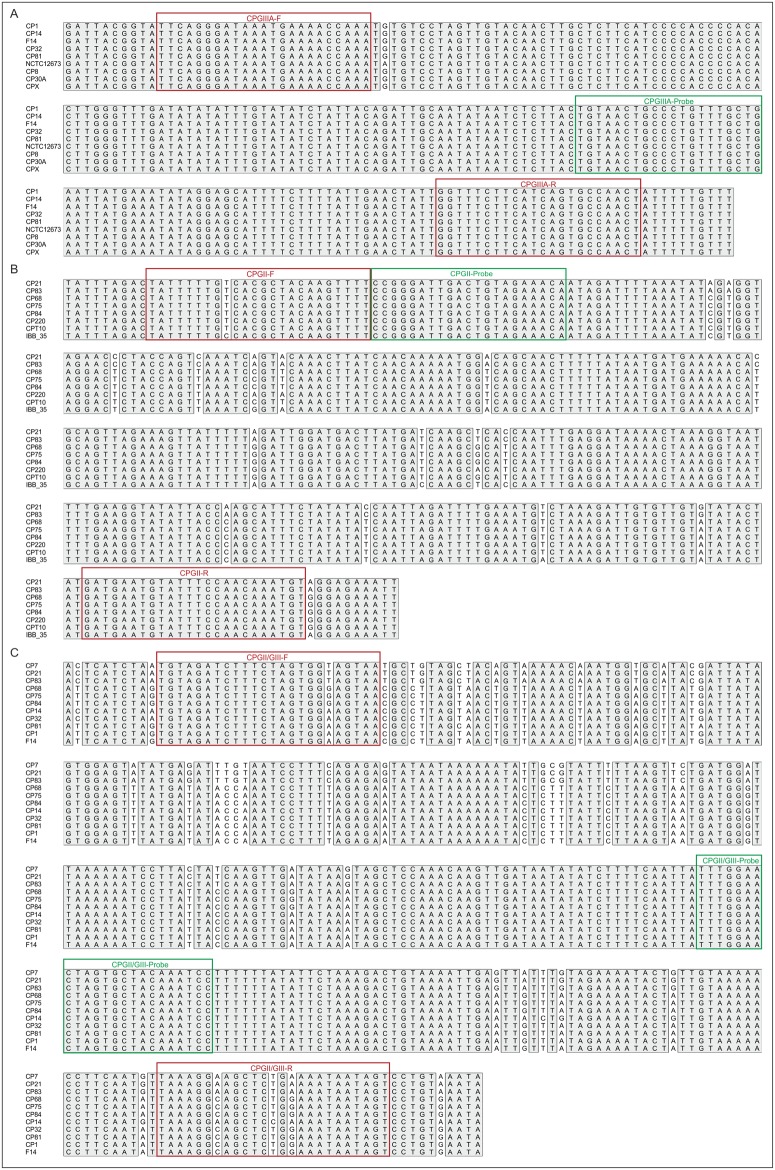
Alignments of target sequences used for the detection of *Campylobacter* phages. Primers and probes for the specific detection of group III phages (A), group II phages (B) and of both groups (C) are boxed by red and green lines, respectively.

To identify nucleotide sequences that are specific for group II or group III *Campylobacter* phages as well as sequences existing in both groups, the genomes of four group II and eight group III phages (Table 1) were compared. Group II and group III phages possess a core genome (Fig 1) comprising genes for virion assembly and proteins, which are important for replication and regulation [26, 27]. Focussing on this core genome, conserved DNA sequences were analysed in detail. For the detection of group II phages, twenty-two possible loci were identified, whereas fourteen potential target candidates were found on the genomes of group III phages (S1 Table). Group II phages generally show only weak DNA similarities to phages of group III but one DNA region within the putative tail fiber gene is conserved in these phages. Primers were deduced from all potential target sequences and examined by conventional PCR (amplicon lengths 800 to 1,500 bp) with our whole collection of *Campylobacter* phages (Table 2). Gel electrophoretic analyses of the amplicons revealed that only few loci were suited to establish a specific PCR detection system for the differentiation of group II and group III phages and for the identification of both groups. Some phages did not show the expected amplicons or the obtained products did not have the predicted sizes. In other cases, several amplicons were produced probably caused by the very low GC content of the phages [23, 25]. Finally, some primers were not specific enough to detect group II or group III phages individually. Nevertheless, for each group, several possible targets were identified. The respective amplicons of all phages were analysed by Sanger sequencing. Unique regions of 150 to 300 bp with an appropriate GC content that were identical or showed only single polymorphisms were used to design primers and probes for real-time PCR. For the detection of group II and group III phages, the tail tube gene (ORF186 of CP21) [29] and the gene for the base plate wedge (ORF008 of CP81) [23], respectively, were selected. Primers (termed “general primers” throughout this publication) derived from the tail fiber gene (ORF096 of CP21 and ORF002 of CP81) were used for the identification of both groups (Figs 1 and 2). The specificity of the primers and probes was confirmed by BLAST searches at the NCBI homepage. All primers and probes were identical or similar to *Campylobacter* phage sequences, whereas they did not match to any other phage DNA, even if up to three mismatches were tolerated. Similarly, none of the primers and probes matched perfectly to any eukaryotic DNA. Initial experiments performed by conventional PCR with DNA of all *Campylobacter* phages demonstrated that the designed primers were suited to obtain the predicted amplicons. The same results were achieved with phage lysates and phages recovered from single plaques. It has been reported that *Campylobacter* phage DNA was refractory to PCR [25]. In this study, DNA of all investigated phages could be amplified using DreamTaq polymerase. We did not test other DNA polymerases, but it might be recommendable to choose the DreamTaq enzyme for the amplification of *Campylobacter* phage DNA.

### Validation of the qPCR and determination of the sensitivity and specificity of the assay

The qPCR assay was validated using lysates of the *Campylobacter* phages CP21 (group II) and CP81 (group III) of the BfR phage collection. Both phages contain the group-specific target region (Tables 1 and 2). Various non-*Campylobacter* phages (NCP, e.g. T1 and T4, phages of other genera) were used as negative controls while two non template controls (NTC) did not contain any DNA. For validation, the assay was performed in triplicate using two different real-time cyclers. The obtained Ct-values (cut-off: Ct ≤ 37.0) indicate that in the presence of *Campylobacter* phages, each of the three primer/probe combinations showed a specific amplification of the respective target sequence with a detection limit of 10 pfu/ml, whereas no amplification was observed with any NCP and the NTC sample. The two real-time cyclers gave similar results (S2 Table). Thus, a reliable detection of *Campylobacter* phages using this assay was independent from the amplification platform. All group II and group III *Campylobacter* phages of the BfR phage collection were detectable with this assay.

### Testing and optimization of the PCR protocol

By testing a 1:10 dilution series of a group II (CP21) and group III (CP81) lysate containing 5 x 10^8^ pfu/ml a detection limit of ~10 pfu/ml was determined (S2 Table). By introduction of an initial heating step (95°C) for 20 min, we were able to decrease the detection limit further by one order of magnitude suggesting that efficient disintegration of the capsids by heat required an extended incubation time. Similar results were obtained by digestion of phage particles with proteinase K/SDS prior to PCR. Table 4 gives an overview about detection limits of conventional and real-time PCR using the designed primers and probes. It can be seen that real-time PCR was two to three orders of magnitude more sensitive than conventional PCR and that the group II- and group III-specific primers allowed a more sensitive detection than the primer pair designed for both groups. The reason for the latter finding might be wobble bases present in the primers targeting the putative tail fiber gene (Fig 2).

**Table 4:** Detection limits of *Campylobacter* phages by conventional PCR and qPCR.

	CPGII detection (Group II-phages)		CPGIII detection (Group III-phages)		CPGII/GIII detection (Both phage groups)	
	PCR	qPCR	PCR	qPCR	PCR	qPCR
CP21 lysate						
Untreated control	10^4^	10^1^	n.d.	n.d.	10^4^−10^3^	10^2^
Heat treatment (20 min, 96°C)	10^3^	10^0^	n.d.	n.d.	10^3^	10^1^
Proteinase K/SDS treatment	10^3^	10^0^	n.d.	n.d.	10^3^	10^1^−10^0^
CP81 lysate						
Untreated control	n.d.	n.d.	10^4^−10^3^	10^1^	10^5^	10^3^
Heat treatment (20 min, 96°C)	n.d.	n.d.	10^3^	10^0^	10^4^	10^2^
Proteinase K/SDS treatment	n.d.	n.d.	10^3^−10^2^	10^0^	10^4^	10^2^

Results are given in pfu/ml.

To test the practicability of the protocol, chicken skin samples were spiked with diluted lysates of group II phage CP21 and group III phage CP81 (see Materials and methods). Upon rinsing of the skin, lytic activity of the phages was quantified by plaque assays. Compared to the phage numbers added to the skin, approximately two orders of magnitude lower titers were determined for recovered phages. Similar results were obtained by PCR indicating that most phages had obviously not been removed from the chicken skin, probably due to a strong adherence to certain surface structures. Since swabbing did not yield more phages, it may be recommendable to stomach chicken skin samples instead of rinsing and swabbing, because this may increase the recovery of phages [8]. In our experiments twice as many phages could be recovered by use of a stomacher. To enhance the sensitivity of the method more significantly, phage particles washed down from skin were concentrated tenfold by centrifugal filter units. By this additional step, the sensitivity could be increased by one log10 unit.

### Detection of *Campylobacter* phages in food and environmental samples

The developed PCR system was applied to detect *Campylobacter* phages in various meat products and samples (water, dust, faeces) collected from chicken and pig farms (Table 5). Altogether 110 samples were analysed of which 65 samples had previously been tested positive for the presence of *Campylobacter*. In 50 samples (45.5%) group II and/or group III phages were identified. The highest incidence rates (57%) were found in poultry and in fecal samples from poultry and swine. Group III phages were twice as frequently found as phages belonging to group II. Overall there was a good agreement between results obtained with the group-specific primers and the general primer pair designed to detect both groups. One exception were two chicken samples which were positively tested with the group II primers but not with the general primers. It is conceivable that these samples contained a novel subgroup of group II phages which may differ from the known subgroups in its tail fiber gene. We recently analysed a set of group II phages isolated in Denmark by PCR. Some phages generated products only with the group II-specific primers. Moreover, restriction patterns of these phages diverged from those of the known subgroups suggesting that they may possess a different modular genome organization [27]. Hence, the combination of the group-specific and general primers allows the detection not only of common, but also of uncommon *Campylobacter* phages which might possess unusual properties and could be suitable candidates for applications.

**Table 5:** Detection of *Campylobacter* phages in environmental and food samples by qPCR.

Sample	No. of samples	CPGII-detection	CPGIII-detection	CPGII/GIII-detection	Negatives
**Meat**	**74 (100%)**	**14 (19%)**	**30 (41%)**	**40 (54%)**	**32 (43%)**
Turkey	19 (100%)	4 (21%)	6 (32%)	10 (53%)	9 (47%)
Chicken	51 (100%)	9 (18%)	22 (43%)	28 (55%)	21 (41%)
Duck	4 (100%)	1 (25%)	2 (50%)	2 (50%)	2 (50%)
**Entrails**	**6 (100%)**	**n.d.**	**n.d.**	**n.d.**	**n.d**
Lamb	2 (100%)	n.d.	n.d.	n.d.	n.d.
Pork	4 (100%)	n.d.	n.d.	n.d.	n.d.
**Milk**	**4 (100%)**	**1 (25%)**	**2 (50%)**	**2 (50%)**	**2 (50%)**
**Feces**	**7 (100%)**	**1 (14%)**	**3 (43%)**	**4 (57%)**	**2 (29%)**
Pig	3 (100%)	1 (33%)	1 (33%)	2 (66%)	1 (33%)
Fowl	3 (100%)	n.d.	2 (66%)	2 (66%)	1 (33%)
Wild birds	1 (100%)	n.d.	n.d.	n.d.	n.d.
**Environment**	**19 (100%)**	**2 (11%)**	**2 (11%)**	**4 (21%)**	**15 (79%)**
Dust & surface	6 (100%)	n.d.	n.d.	n.d.	6 (100%)
Water	13 (100%)	2 (15%)	2 (15%)	4 (31%)	9 (69%)
**Total**	**110 (100%)**	**18 (16%)**	**37 (34%)**	**50 (45%)**	**51 (46%)**

n.d., not detected

Two samples (one chicken leg and one fecal sample of swine) gave products with the general primer pair but not with the group II- and group III-specific primers. The reason for this result is yet not clear but it cannot be excluded that the samples contained *Campylobacter* phages which do not belong to group II or III. The fact that phages exhibiting lytic activity on the used indicator strains could be isolated from only one out of 110 samples suggests that some samples may have contained *Campylobacter* phages with an uncommon host range or that the number of phages was very low In addition, it has to be taken into account that most samples were stored frozen, which may have had a negative effect on the lytic activity of the phages [8].

Our study showed that the majority (52%) of the 65 *Campylobacter*-positive samples contained group II or group III phages whereas only 38% of the remaining 45 samples whose *Campylobacter* content was unknown, were phage-positive. In another study *Campylobacter* phages were isolated from 34/300 (11%) chilled retail chicken portions by plaque assays. Phages were exclusively recovered from samples that also harbored *Campylobacter* [8]. These findings demonstrate that on the one hand *Campylobacter*-specific phages can be readily found at locations where their hosts occur, but that on the other hand the presence of those phages is a good indicator for *Campylobacter*. The data also show that *Campylobacter* phages can be detected with much greater sensitivity by PCR than by plaque assays and that the molecular approach can help to identify phages that might possess novel properties, e. g. an uncommon host range. However, it should be emphasized that although the use of two primer/probe sets allows a more reliable detection of *Campylobacter* phages than a single set, it cannot be ruled out that other yet unknown phages may cause false-positive results. Since both active and inactivated phages are detected by PCR, a combination of this technique with plaque assays is the most promising strategy to identify and isolate new *Campylobacter* phages.

## Conclusions

Phages are a promising tool to reduce *Campylobacter* along the food chain. Though, some phenotypic and genotypic properties of *Campylobacter* phages make it rather difficult to deal with them. The multiplex real-time PCR developed in this study may help to overcome some of the problems. First and foremost, the PCR system allows a quick and sensitive detection and discrimination of group II and group III phages. It can be used to pre-screen even large numbers of samples possibly containing *Campylobacter* phages. Due to the high sensitivity of the PCR system, negative samples are not very likely to harbor phages belonging to one of these groups. On the other hand, PCR-positive samples, which did not show lytic activity on common indicator strains could be tested with other strains that may be suitable hosts for group II or group III phages. The PCR system can also be used in host phage interaction studies, where even low numbers of active and inactive phages can be determined very quickly. Finally, it can be included in metagenomic analyses of phage communities, as it facilitates the detection of *Campylobacter* phages containing modified DNA.

## Supporting information

S1 Table Common genes of group II and group III phages and primers deduced from these targets. (DOCX)

S2 Table Group II and group III phage detection on different real-time platforms. (DOCX)
