# Supplementary material for: A multiplex real-time PCR for the detection and differentiation of Campylobacter phages
Source: PLoS One. 2017 Dec 22;12(12):e0190240. doi: 10.1371/journal.pone.0190240 (PMC5741259; doi:10.1371/journal.pone.0190240)
Supplement: S1 Table — (DOCX) [file pone.0190240.s001.docx]

**Supporting information**

S1 Table: Common genes of group II and group III phages and primers deduced from these targets.

| **Oligonuceotide** | **Sequence (5‘-3‘)** | **Position on genome** | **CDS** |
| --- | --- | --- | --- |
| **CP220likevirus phages** | | | |
| JCY001-F | CGGTGCTGTATCTCCAGTTG | CP220 (ORF0051) | Possible major capsid protein |
| JCY001-R | TTTGGAAGCTTAATGGCA | 47099-47985 |  |
| JCY002-F | CTGAAGCCATTGTTGAAGCA | CP220 (ORF0053) | Possible phage tail sheath protein |
| JCY002-R | GACCCCTGTTAAGACCAGCA | 50211-51154 |  |
| CP220_3-F | TCGATGGATCTACGCAAGAA | CP220 (ORF0080) | Hypothetical radical SAM protein |
| CP220_3-R | GGTGCACACCAACGTGTATT | 71009-71470 |  |
| CP220_4-F | GATTTGCGACAGATGGGTCT | CP220 (ORF0083) | Hypothetical radical SAM protein |
| CP220_4-R | TCCAAGAAGTTGAAAGCAAACTC | 73734-74132 |  |
| CPt10_6-F | AACAGACCCACCATACTTCG | CPt10 (ORF1471) | Possible phage DNA methylase |
| CPt10_6-R | TTTTTGTGTTGGGTGAACGA | 123845-124234 |  |
| CPt10_7-F | TCCCAAATATTGAGAGCGATT | CPt10 (ORF0091) | Type III Modification enzyme |
| CPt10_7-R | ATGCCTATCCATTTGCGTTT | 8509-8959 |  |
| CP220_11-F | GCCTTGGTTTTCGGCTACTT | CP220 (ORF0138c) | ISCaje3 transposase |
| CP220_11-R | TGCTGGAGCAAAAATAGAAGG | 123665-124256 |  |
| CP220_12-F | GGTGAAGCATCTAAGAGTGGAAA | CP220 (ORF0146) | ISCaje4 transposase TnpB |
| CP220_12-R | TCTTTGGCTCCTTGCTTCAT | 128795-129357 |  |
| CPt10_13-F | GGGGTGAACCCACATTGTT | CPt10 (ORF0871) | Hypothetical radical SAM protein |
| CPt10_13-R | CCAAGCTTTTAGATCCAACTTCA | 74106-74642 |  |
| CPt10_14-F | CTTGTGCAATATGCCCACAC | CPt10 (ORF0931) | Hypothetical radical SAM protein |
| CPt10_14-R | AAACGTTTTTCCCAATCGTG | 78693-79263 |  |
| CPt10_17-F | GTACCAAGTTCGGACGCATT | CPt10 (ORF0001) | Putative phage DNA packaging protein (terminase) |
| CPt10_17-R | CAGCAACCACTTGACCAGAA | 1146-1857 |  |
| CPt10_19-F | CAGGAAAAACAAGCACAGCA | CPt10 (ORF0101) | Putative sliding clamp loader |
| CPt10_19-R | TCTCTTGAGAAAGCACTTTGG | 9713-10424 |  |
| CPt10_20-F | TGCTGAATGTTGGAGTGGTT | CPt10 (ORF0291) | Putative portal vertex protein |
| CPt10_20-R | GCATACCTTTAGCACCAGCTTT | 23800-24569 |  |
| CPt10_21-F | AACACCGGAAATAACCAGGA | CPt10 (ORF0321) | Possible phage DNA ligase |
| CPt10_21-R | CGCTAATCCCAGAAACTTTACC | 26269-27077 |  |
| CPt10_24-F | GAGTGAAACTCGTATGAAACACG | CPt10 (ORF0461) | Putative sigma factor for T4-like late transcription |
| CPt10_24-R | CTCAGAACATTTTTAAGTTCAGCA | 44478-45194 |  |
| CPt10_25-F | TCAGCTCCAGGTATTGACGA | CPt10 (ORF0511) | Possible phage tail sheath protein |
| CPt10_25-R | GGCGATTACACTTGCTGGAT | 50470-51231 |  |
| CPt10_26-F | TCAAGTACTAGGTGATTTAGCTCGTC | CPt10 (ORF0431) | Putative tail tube protein |
| CPt10_26-R | TGAATCTGAAACCGAATCCA | 41369-42071 |  |
| CP220_27-F | TTCGGTTCTGTGTTCTCCAC | CP220 (ORF0054c) | Putative Hef like homing endonuclease |
| CP220_27-R | CGTAACAGCCGCAAAATACA | 51865-52608 |  |
| CP220_39-F | GGCTCCTGCAGGTATTGGTA | CP220 (ORF0125) | Possible phage ATP-dependent primase-helicase |
| CP220_39-R | GGAACTTCTGCTGGAGCTTG | 111324-111633 |  |
| CP220_47-F | GGAATCACGCTAAAGCAGGT | CP220 (ORF0005) | Putative phage DNA topoisomerase (large subunit) |
| CP220_47-R | GGACACCACCGTCTGGTATT | 4215-4721 |  |
| CPt10_48-F | GATGGTGGAGCTTACGAAGG | CPt10 (ORF0391) | Possible phage baseplate wedge protein |
| CPt10_48-R | TGGTGCATTTGTTTTGATGG | 32134-32970 |  |
| CP220_51-F | AGGGGGCTTTGTAGCTGAAC | CP220 (ORF0115) | Possible phage DNA polymerase |
| CP220_51-R | AGAGCTTGCAATCGCTTCAC | 102964-103813 |  |

CP81unalikevirus phages

| **Oligonuceotide** | **Sequence (5‘-3‘)** | **Position on genome** | **CDS** |
| --- | --- | --- | --- |
| CP81_019-F | ATAGAAGAAAATGGGCTTGAGG | CP81 (ORF008) | Putative baseplate wedge protein |
| CP81_019-R | AGTCATCCGAACTTATTTGTGG | 7898-8999 |  |
| CP81_099-F | GGGCTAACACCAGGACTAAC | CP81 (ORF049) | RecA-like recombination protein |
| CP81_099-R | ACTAAAGAACTCATCAAGCTGC | 39595-40392 |  |
| CP81_113-F | TGCCTGAGCATAATCAAAGAC | CP81 (ORF054) | Putative ribonucleotide reductase, small subunit |
| CP81_113-R | GGCAACATTTCCAATCTCTCC | 43162-44099 |  |
| CP81_115-F | TGGAGAGATTGGAAATGTTGC | CP81 (ORF055) | Putative ribonucleotide reductase, large subunit |
| CP81_115-R | TGAACATCCATATGCCATGTTG | 44078-45041 |  |
| CP81_189-F | ATGATTCAGCACCACTTGTAG | CP81 (ORF103) | Putative major head protein II |
| CP81_189-R | CAACCACAACAGTTGATATTGC | 67541-68386 |  |
| CP81_220-F | AGGTGGAAACCCTGATGAC | CP81 (ORF119) | Putative DNA primase subunit |
| CP81_220-R | CCACATCCTCAATTTGCATTC | 79376-79913 |  |
| CP81_249-F | CGTTCAATGTGATGATAACGAG | CP81 (ORF133) | Putative adenine specific DNA methyltransferase |
| CP81_249-R | GGTATTCAAAATCTCCACCACC | 87767-88602 |  |
| CP81_259-F | ATGTGTAATGGCAACTGGTAG | CP81 (ORF144) | Putative DNA helicase UvsW |
| CP81_259-R | TGCATATCTTCCTCTAGCGTC | 93573-94617 |  |
| CP81_286-F | TCAGTTGTTTCAGAATCACCG | CP81 (ORF154) | Putative phage DNA packaging protein |
| CP81_286-R | GTCAGATGCTGTTGAAGGTAAG | 101952-102779 |  |
| CP81_290-F | ACCCACATAAAGAAGCCAAC | CP81 (ORF157) | Putative portal vertex protein |
| CP81_290-R | ATTGGATTCCTTTTCCCAACC | 104799-105979 |  |
| CP81_298-F | TGAGAACTGTGCTTGAAAACC | CP81 (ORF161) | Putative major capsid protein |
| CP81_298-R | GTGCAAAGAAAATACCAGCATC | 108093-109238 |  |
| CP81_317-F | AATGAGAATACAGATGCAGGC | CP81 (ORF171) | Putative DNA ligase |
| CP81_317-R | AACCAGTTCCAACACATACTTC | 115810-116902 |  |
| CP81_355-F | TTGCTTTGGAGGGATCTAATTC | CP81 (ORF186) | Putative RNAseH |
| CP81_355-R | TCTCTTAGCCCCTATTTCACC | 130474-131051 |  |
