# Supplementary material for: A multiplex real-time PCR for the detection and differentiation of Campylobacter phages
Source: PLoS One. 2017 Dec 22;12(12):e0190240. doi: 10.1371/journal.pone.0190240 (PMC5741259; doi:10.1371/journal.pone.0190240)
Supplement: S2 Table — (DOCX) [file pone.0190240.s002.docx]

**Supporting information**

**S2 Table. Group II and group III phage detection on different Real-Time plattforms.**

|  | **ABI 7500 Fast Real-Time PCR System (Applied Biosystems)** | | | **CFX96 Real-Time PCR System**  **(Bio-Rad Laboratories GmbH)** | | |
| --- | --- | --- | --- | --- | --- | --- |
|  | CPGII | CPGIII | CPGII/III | CPGII | CPGIII | CPGII/III |
| **NCP** | **n.d.** | **n.d.** | **n.d.** | **n.d.** | **n.d.** | **n.d.** |
| T1 | n.d. | n.d. | n.d. | n.d. | n.d. | n.d. |
| T4 | n.d. | n.d. | n.d. | n.d. | n.d. | n.d. |
| **NTC** | **n.d.** | **n.d.** | **n.d.** | **n.d.** | **n.d.** | **n.d.** |
| NTC | n.d. | n.d. | n.d. | n.d. | n.d. | n.d. |
| **CP21** | **+** | **-** | **+** | **+** | **-** | **+** |
| ~10^5^ | 23.4 (±0.545) | n.d. | 25.0 (±0.733) | 24.1 (±0.339) | n.d. | 25.2 (±0.639) |
| ~10^4^ | 27.8 (±0.488) | n.d. | 28.7 (±0.549) | 27.5 (±0.274) | n.d. | 28.0 (±0.445) |
| ~10^3^ | 30.5  (±0.601) | n.d. | 31.6 (±0.561) | 30.9  (±0.508) | n.d. | 31.7  (±0.726) |
| ~10^2^ | 34.7  (±0.823) | n.d. | 35.5 (±1.004) | 34.0 (±0.777) | n.d. | 34.7 (±0.565) |
| ~10^1^ | 36.3  (±1.317) | n.d. | 37.4  (±1.167) | 36.5 (±1.201) | n.d. | 36.3 (±1.087) |
| ~10^0^ | 38.1  (±1.799) | n.d. | 37.9 (±2.020) | 37.7  (±1.337) | n.d. | 38.5  (±1.474) |
| **CP81** | **-** | **+** | **+** | **-** | **+** | **+** |
| ~10^5^ | n.d. | 21.5 (±0.389) | 22.2 (±0.483) | n.d. | 22.6 (±0.367) | 23.8 (±0.563) |
| ~10^4^ | n.d. | 25.2 (±0.667) | 26.3 (±0.611) | n.d. | 25.1 (±0.203) | 27.8 (±0.462) |
| ~10^3^ | n.d. | 29.0  (±0.549) | 30.1  (±0.847) | n.d. | 29.9  (±0.448) | 31.5  (±0.536) |
| ~10^2^ | n.d. | 33.1  (±0.899) | 34.0  (±0.992) | n.d. | 34.3  (±0.658) | 34.6  (±1.005) |
| ~10^1^ | n.d. | 36.8  (±0.936) | 37.3  (±1.255) | n.d. | 37.0  (±1.056) | 37.9  (±1.674) |
| ~10^0^ | n.d. | 38.3  (±1.554) | 39.7  (±1.832) | n.d. | 39.4  (±1.980) | 40.1  (±2.314) |
